# History of ocular plastic surgery in Brazil - Memories

**DOI:** 10.5935/0004-2749.20210119

**Published:** 2025-08-21

**Authors:** Eduardo Jorge Carneiro Soares

**Affiliations:** 1 Departamento de Oftalmologia, Faculdade de Medicina, Universidade Federal de Minas Gerais, Belo Horizonte, MG, Brazil

## THE BEGINNINGS OF OCULAR PLASTIC SURGERY

The Code of Hammurabi of the King of Babylon (2250 BC) provides the first reference to ocular plastic surgery. From the time of Hippocrates (460-370 BC) to the nineteenth century, there are only two noteworthy reports of blepharoplastic-cosmetic reconstructions in the eyelid region made by Celsus AC (25 BC to 50 AD) and Ambroise Paré (1509-1590). The treatment of orbital and eyelid lesions remained outdated until the beginning of the Contemporary Age when the works of Von Graefe (1787-1840), a pioneer of ophthalmology and creator of several facial plastic surgeries, were published in 1818. He was later followed by Dieffenbach JF (1792-1847), the author of several procedures for strabismus and reconstruction of the lower eyelids that were published in 1829. Dr. Von JF Dieffenbach is generally regarded as the father of plastic surgery.

## THE BIRTH OF MODERN OCULAR PLASTIC SURGERY

“War is the only proper school for surgeons,” Hippocrates.

Plastic surgeons have been influenced by the horrors of the two world wars (1914-1918; 1939-1945) and were overwhelmed and unable to deal with the thousands of mutilated patients. The orbital and eyelid lesions were referred to eye surgeons at that time. The demands and challenges of World War I forced John Martin Wheeler (1879-1938) to develop reconstructive surgical techniques that were published in 1920^(1)^. Wheeler was the first to provide courses to teach ophthalmic plastic surgery and earned the title “Father of Ophthalmic Plastic Surgery.”

During World War II, when ocular plastic surgery was recognized as a subspecialty of ophthalmology in the United States, Wendell L. Hughes (1900-1994), one of Wheeler’s students, improved oculoplastic surgery not only in publications for disseminating his experiences but also by teaching and training several famous surgeons. In 1969, he founded the American Society of Ophthalmic Plastic and Reconstructive Surgery (ASOPRS) and was its first president.

In Britain, World War II allowed Hyla B. Stallard (1901-1973) to play the same role in pioneering and significantly contributing to the progress of oculoplastics. Among his illustrious followers, it is worth mentioning John Clark Mustardé, one of the pillars of modern oculoplastic surgery. Mustardé exercised significant influence on the foundation of the Brazilian society.

## HIGHLIGHTS DURING PRE AND POST WORLD WAR II IN BRAZIL

### JOSÉ LOURENÇO DE MAGALHÃES

Born in Sergipe, Brazil, José Lourenço de Magalhães graduated as a Doctor of Medicine at the pioneering Medical School of Salvador, Bahia. As member of the Imperial Academy of Medicine, he also authored the first publication concerning the correctness of an eyelid deformity, viz., “Surgery of ectropion,” which was published by the Medical Gazette of Bahia (1864).

### DONATO VALLE

Born in Varginha, Brazil, and graduated in otolaryngology, Donato Valle honed his surgical expertise at the Penido Burnier Institute. He presented his first work at the first Brazilian Congress of Ophthalmology (“Dacryocistite and its treatment”) in 1935. He perfected the technique of transcutaneous dacryorhinostomy described by Dupuy Dutemps, posting his procedure in the Arquivos Brasileiros de Oftalmologia, vol. 3:101-125, 1940. He also contributed with new tools that actually facilitated the execution of the procedure.

Valle innovated surgery of the lacrimal system in Brazil, publishing several works in the 1930s and 1940s.

### IVO HÉLCIO JARDIM DE CAMPOS PITANGUY

Born in Belo Horizonte, Brazil, Ivo Pitanguy is considered as the most renowned plastic surgeon in Brazil. He graduated in medicine in 1946, spending the first years working in the most famous plastic surgery centers in the world. He acquired extensive knowledge and experience. Returning to Brazil in the late 1940s, a time when plastic surgery was not yet recognized as a medical specialty, he created the service in the Santa Casa de Misericórdia Hospital in Rio de Janeiro. There he began to train national and foreign experts and taught training courses including in the eyelid area. His book, “Atlas of Eyelid Surgery,” published in 1994, is an indicator of his experience and attention to the importance of the orbital-palpebral area in the context of esthetics and facial beauty.

## INFLUENCES ON THE EMERGENCE OF OCULOPLASTIC SURGERY IN BRAZIL

### BYRON CAPLEESE SMITH

Byron Smith was the Director of the Department of Ophthalmology at New York Medical College and Emeritus Chief Surgeon of the Division of Ophthalmic Plastic Surgery from Manhattan Eye, Ear and Throat Hospital. He was one of Hughes’ disciples during World War II. Together, they founded the first clinic entirely devoted to oculoplastic surgery at New York University in 1941. His studies on the mechanism of orbital fractures helped systematize the treatment of orbital trauma. Dr. Smith answered to several calls of Brazilian ophthalmology to minister courses and lectures at meetings and was always forthcoming to all who wanted his internships and teachings.

### BERNARD A. WEIL

Bernard Weil was one of the pillars of dacryology. Alongside Benjamin Milder, he published the book “The Lacrimal Syeestem” in 1983, which is considered as one of the most important works on the pathology and surgery of the lacrimal system. He began contributing to the training of Brazilian ophthalmologists in October 1976, after his conference “Propedeutics of the Lacrimal System” held in the 3rd Meeting of the Center of Studies on Oculoplastic in Rio de Janeiro. Several experts, including Eduardo Soares in 1977 and Marilisa Nano Costa in 1980, have had the privilege of doing internships under his guidance and to be at his service at the Hospital de Niños and at the Centro Privado de Ojos in Buenos Aires (Argentina). All are grateful for the teachings transmitted in Weil’s courses, lectures, and conferences in our country.

### JOHN CLARK MUSTARDÉ

Mustardé began practicing medicine as an ophthalmologist. However, during World War II, he worked alongside the great masters in the field of general plastic surgery, having also acquired the title of expert in this area. He is admittedly one of the most important surgeons of the twentieth century in medical practice, having been a pioneer in various procedures. His important contributions to ophthalmic plastic surgery, especially his techniques of eyelid reconstruction, must be particularly emphasized.

In the teaching field, Professor Mustardé taught with great prominence, spreading disciples to several countries, such as in Canniesburn Hospital in Glasgow, Scotland. His book, “Repair and Reconstruction in the Orbital Reegion,” 1966, is a real bible for ocular plastic surgeons. Moreover, he taught the exercise of Hippocratic and humanitarian medicine, seeking perfection with humility and perseverance.

In the associative area, he was the founder of several entities such as the European Society of Ocular Plastic Surgery. Several honors and laurels were granted to him, including the title of “Sir” by the Queen of England.

He was present at the foundation of the Center of Studies of Oculoplastic Surgery, which happened during a reunion of the Brazilian Ophthalmological Society in Rio de Janeiro, November 27, 1974. He was given the title of Honorary President. The author thanks his contributions and teachings on behalf of Brazilian Ophthalmology.

### HILTON ROCHA

**Figure 1:**
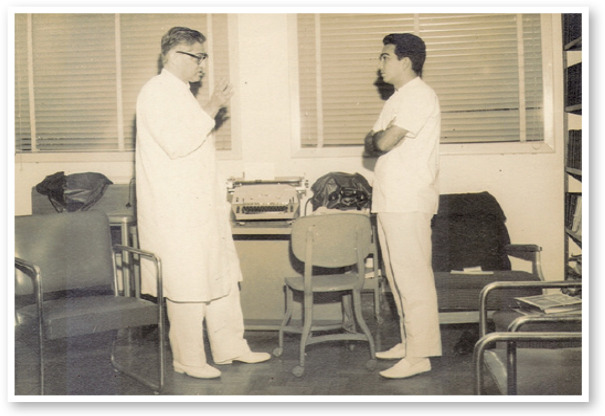
1966: Professor Hilton Rocha and Eduardo Soares - in charge of the Oculoplastic Service - at the library of São Geraldo Hospital, UFMG.

Hilton Rocha was the Chairman of the Department of Ophthalmology, Faculty of Medicine, at the Federal University of Minas Gerais (Hospital São Geraldo) and was responsible for the creation (in 1959) of the first specialization course in ophthalmology in Brazil. Until then, ophthalmology was not divided into sectors but was taught by teachers according to their personal and professional experiences without a systematic program of teaching. He implemented various sectors of specialty, initially contemplating glaucoma, strabismus, retina, uveitis, contact lenses, and patholoegy. The first group of ophthalmologists graduated in 1961. Since then, this model of education began to be adopted by other institutions throughout Brazil. In 1966, foreseeing the future of the specialty, Professor Hilton Rocha created the Sector of Ocular Plastic Surgery, a pioneer in Brazil, and placed the young Eduardo Jorge Carneiro Soares in charge (Figure 1). That was how ocular plastic surgery began to be taught and added to the training of Brazilian’s ophthalmologists.

## OCULOPLASTIC SURGERY IN BRAZIL 1966-1974: THE FIRST FELLOWSHIP THE BIRTH OF THE BRAZILIAN SOCIETY

The newly created Sector of Ocular Plastic Surgery began its activities in 1966, working on the 3rd floor of Hospital São Geraldo in a very small room. It had a spotlight along with a file for medical records and slides. Photodocumentation was made using an Asai-Pentax camera equipped with a macro lens and ring flash. There was only a simple wooden chair where the patient sat, another one for the examiner, and a small table. The resident stood around.

**Figure 2:**
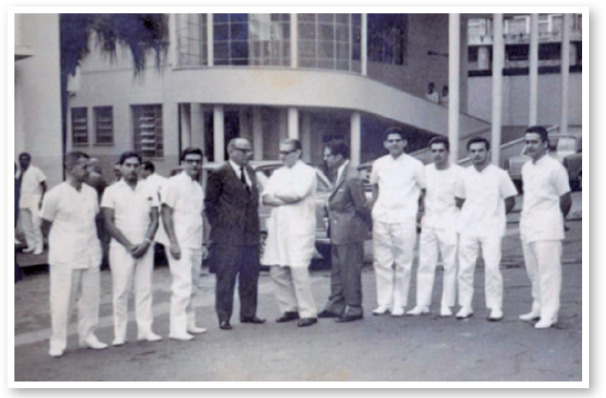
1967: The seventh group of residents (1965-1967) was the first to receive teaching in oculoplastic surgery during the Specialization Course of Ophthalmology in Brazil, São Geraldo Hospital, UFMG.

During this period, in addition to patient care, the author taught the theoretical program and attended outpatients and surgical patients from the residents of the Specialization Course in Ophthalmology, which was conducted in the second year of the course in a 2-month rotation. The seventh group of residents was the first to receive teaching in oculoplastic surgery (Figure 2).

The resident completed the internship with a basic understanding of the specialty. The subject of oculoplastic surgery was then established in the Ophthalmologist Training Course and served as an example to other university residences in Brazil to incorporate it in their teaching programs.

**Figure 3:**
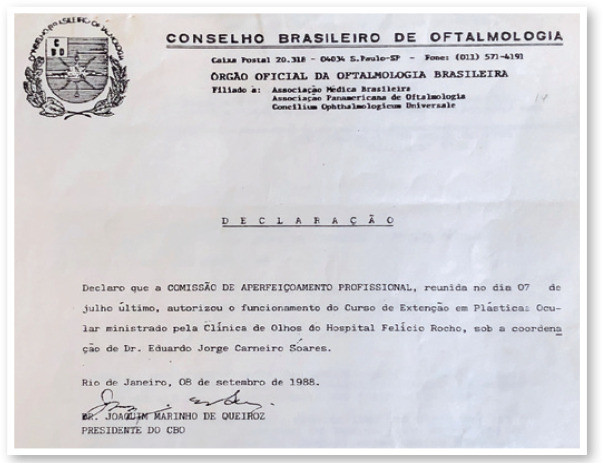
The Fellowship was recognized by the Brazilian Council of Ophthalmology with the name of Extension Course in Oculoplastic Surgery.

Furthermore, the Sector produced scientific publications, courses, lectures, and presentations at congresses and meetings in Brazil and abroad, thus promoting the specialty. In 1971, after his stay in Canniesburn Hospital in Glasgow (Scotland), where Eduardo Soares worked with Professor John C. Mustardé, oculoplastics specialization was created in the Fellowship system, with exclusive dedication for a year. The first Fellow was Alfredo Bonfioli (04/01/74 to 03/31/75). Hence, this course was born, a pioneer in Brazil, and it has produced oculoplastic surgeons every year. It was recognized in 1988 by the Brazilian Council of Ophthalmology with the name of Extension Course in Oculoplastic Surgery (Figure 3).

Evaldo Santos, Eloy Pereira, and Eduardo Soares were the three doctors responsible for creating a Center of Study to teach and promote ocular plastic surgery in Brazil. They began to discuss the issue after presenting lectures in the same scientific session of the Brazilian Congress of Ophthalmology in Porto Alegre (RS) in 1969. Back then, there was only the Brazilian Center for Strabismus.

### AND SO OUR SOCIETY WAS BORN (1974)

**Figure 4:**
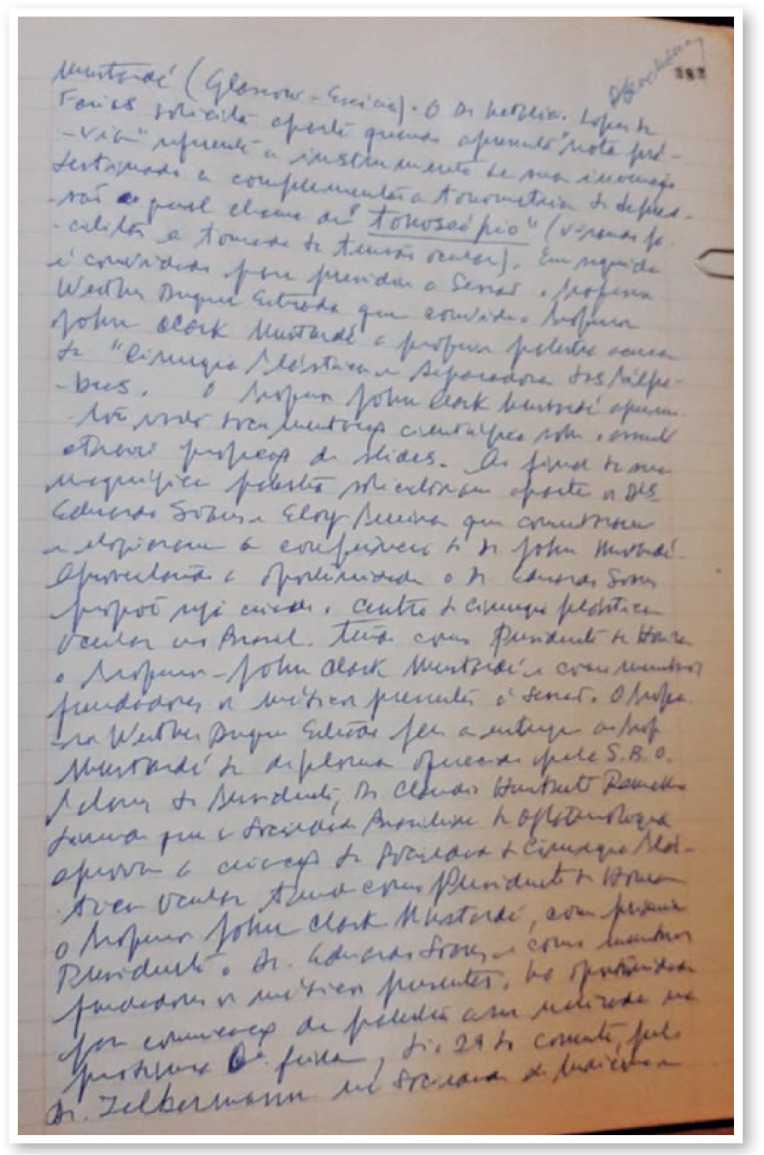
1974: Historical document. The pages 382-384 of the Minutes Book of the Brazilian Society of Ophthalmology register the foundation of our Society with the name “CENTRO DE ESTUDOS DE PLÁSTICA OCULAR” - CEPO (Study Center of Oculoplastics), on November 21, Rio de Janeiro.

**Figure 5:**
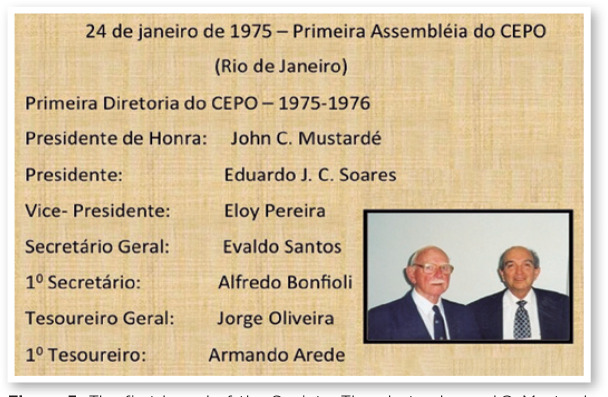
The first board of the Society. The photo shows J.C. Mustard and Eduardo Soares.

A significant incentive for the trio was given by Jack Mustardé in October 1971 when he visited Brazil invited by the Brazilian Portuguese-Spanish Congress in Rio de Janeiro (RJ). The statutes were prepared and, on November 21, 1974, they founded the society with the name “CENTRO DE ESTUDOS DE PLÁSTICA OCULAR” - CEPO (Study Center of Oculoplastics), at the head office of the Brazilian Society of Ophthalmology in RJ (Figure 4). The statutes contemplated the aim of bringing together ophthalmologists interested in the field to share knowledge and develop ocular plastic surgery in Brazil. Professor John Clark Mustardé, present at the meeting, was awarded unanimously the title of Honorary President. Eduardo Jorge Carneiro Soares was awarded the President of the first Board of Directors for a 2-year term (Figure 5).

## PIONEERS OF CENTRO DE ESTUDOS DE PLÁSTICA OCULAR - CEPO

### EVALDO MACHADO DOS SANTOS (1916-1999)

From Jaquarão (RS/Brazil), Evaldo Machado dos Santos graduated from the Federal University of Rio Grande do Sul in 1941. In ophthalmology, he was a student of Professor Ivo Correia Meyer. He was a specialist in strabismus and also dedicated himself to oculoplastics and honed his skills under the guidance of Professor Byron Smith in 1951 in New York (USA). He exercised his activities at the Red Cross Hospital in RJ. He created the Ophthalmology Service of the Air Force Hospital of RJ. His lectures were preferably about ptosis and marginal deformities, areas in which he had great experience.

### SEBASTIÃO ELOY PEREIRA (1936-2017)

He was born in Taubaté (SP), on the day of Saint Sebastian, January 20, 1936, a fact that explains the origin of his name. He graduated in medicine at the University of Medical Sciences, RJ, and did his residence at the Department of Ophthalmology under the guidance of Professor Werther Duque Estrada. In oculoplastics, he was a student of John Clark Mustardé in Ballochmyle Hospital, Scotland, in 1966. He returned to work under the service of Professor Werther Duque Estrada in RJ. In November 1967, he moved to Campo Grande (MS/Brazil), Mato Grosso do Sul (UFMS), to head the Department of Ophthalmology at the Federal University of Mato Grosso do Sul (UFMS). He excelled in eyelid reconstruction techniques, which were taught by Jack Mustardé. He was a very skilled surgeon in reconstructive plastic surgery of the eyelids and orbital region. He suspended these activities in 2013 due to health reasons. The theme earned him courses, lectures, and publications.

### EDUARDO JORGE CARNEIRO SOARES

Born in Belém (PA/Brazil) on October 5, 1938, Eduardo Jorge Carneiro Soares graduated at the Medical School of the Federal University of Pará in 1962. After graduation, he joined the fifth class of the Specialization Course in Ophthalmology at the Medical School of the Federal University of Minas Gerais (under the service of Professor Hilton Rocha) in São Geraldo Hospital. Upon receiving the title of specialist in ophthalmology in 1965, he was invited to the honorable mission of joining the faculty of the course as head of the newly created Sector of Ocular Plastic Surgery. He raised the banner “Learning and Teaching” that still flies to this day in his professional routines. In 1971, he attended the Department of Plastic Surgery of Professor Jack Mustardé in Canniesburn Hospital in Glasgow (Scotland), where he honed his skills. Upon returning, he created in São Geraldo Hospital the first year-long course of Fellowship in ocular plastic surgery with exclusive dedication. From the beginning of his activities, Dr. Soares dedicated special attention to mutilated patients by anophthalmic cavities. At that time, Brazilian surgeons did not use implants to replace the ocular volume, thus condemning patients to suffer physically and emotionally the hardships of deformities inherent to empty anophthalmic sockets. There have been several classes, lectures, and publications to change this situation. This culminated in the doctoral thesis “The Importance of anatomical and functional Reconstruction of the anophthalmic cavity in the Prevention and Treatment of the retraction process of the conjunctival fornices,” approved at UFMG on August 28, 1992. The victory of this struggle was happily achieved. In Brazil, it is currently rare patients who fail to have their orbits recovered in the same act of enuclea tion or evisceration.

**Figure 6:**
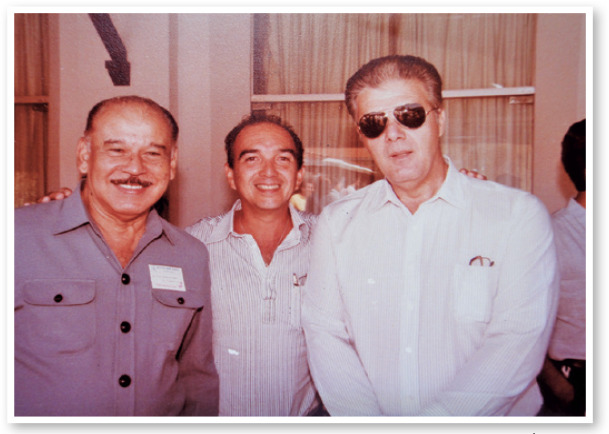
1974: The founders of the CENTRO DE ESTUDOS DE PLÁSTICA OCULAR: *Evaldo M. Santos, Eduardo J. C. Soares*, and *Sebastião Eloy Pereira.*

**Figure 7:**
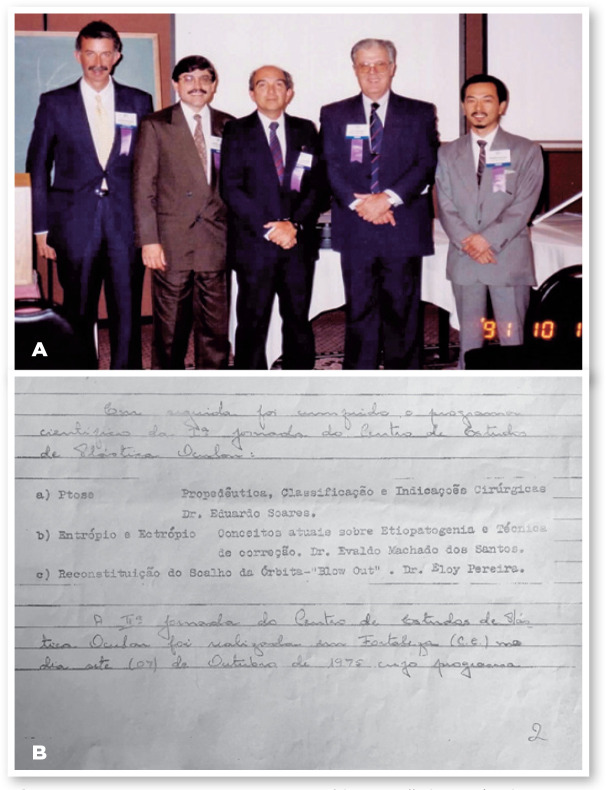
A. In the first CEPO meeting: Waldyr Portellinha, Eurípedes Moura, Eduardo Soares, Eloy Pereira, and Henrique Kikuta. B. 24.01.1975. The first CEPO meeting scientific program.

Initially with only the three founders (Figure 6) and along with a few faithful companions, CEPO was not an improvised work and neither emerged as an ephemeral boost of its founders’ aspirations. Among those companions from the first days who devoted themselves to the institution and contributed to its progress (Figure 7 A and B), some of the following people deserve special attention:

Armando Arede

Cássio Galvão Monteiro

Eurípedes Mota de Moura

Henrique Kikuta

Jaime Roizenblatt

Janduhy Perino Filho

Jorge Alberto de Oliveira

José Aparecido Deboni

José Daphnis Mil Homens Costa

José Vital Filho

Luiz Augusto Morizot Leite Filho

Marcos Cunha

Marilisa Nano Costa

Mário Luiz Monteiro

Mário Perez Genovesi

Mauro Rabinovith

Paulo Goes Manso

Roberto Abuchan

Roberto Caldato

Vicente Muniz de Carvalho

Waldyr Martins Portellinha

Zeniro José SanMartin

### 1979 - CHANGING THE NAME CEPO TO SBCPO

In 1979, a change in the legal situation of CEPO was a necessity to allow its integration along with the other affiliated societies to the Brazilian Council of Ophthalmology. The CENTRO DE ESTUDOS DE PLÁSTICA OCULAR (CEPO) became the SOCIEDADE BRASILEIRA DE CIRURGIA PLÁSTICA OCULAR (SBCPO) on September 8, 1979, in the General Assembly of CEPO held in São Paulo (SP) during the XX Brazilian Congress of Ophthalmology.

**Figure 8:**
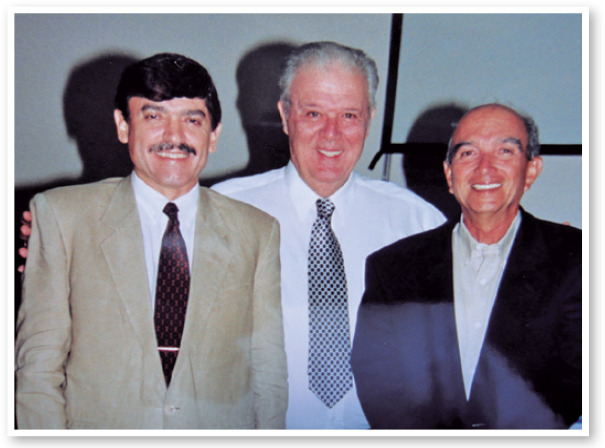
Eurípedes Mota de Moura, Eloy Prereira, and Eduardo Soares. Eurípedes changed the legal identity of the Study Center to Brazilian Society.

It was granted to the board of directors chaired by Eurípides da Mota Mourae, and with Waldir Martins Portellinha as the secretary, the Study Center was transformed into a legally constituted entity named the Brazilian Society of Ocular Plastic Surgery (SBCPO) (Figure 8). The statutes of the Association were registered under No. 16,727, in the 3rd Civil Registry of Legal Entities of São Paulo (SP).

The legal regularization of CEPO, transforming its identity into a new institution, allowed the Society to request its integration along with the other affiliated societies to the *Conselho Brasileiro de Oftalmologia.* This was formalized on October 22, 1981, under the management of the second term of President Eduardo J. C Soares. Thus, oculoplastic surgery was recognized by the *Conselho Brasileiro de Oftalmologia* as a subspecialty of ophthalmology.

### 1997 - THE BOOK

On September 3, 1997, the book “Oculoplastic Surgery” - Roca Publishing, São Paulo/SP - was launched in Goiânia (GO) in the presence of Professors J.C. Mustardé and Richard Collin. This was the main theme of the XXIX Brazilian Congress of Ophthalmology. The editors were Professors Eduardo J.C. Soares, Eurípides M. Moura, and João Orlando R. Gonçalves. The book was the conclusion of the work done since 1974, when the Study Center of Oculoplastics (CEPO) was founded. The knowledge acquired by collaborators through lectures, courses, symposia, and publications made during all these years were expressed and disseminated in that book. It brought together the experience and lessons of all those who participated in the activities of the Brazilian Society of Oculoplastic Surgery. Being sold-out, the book has been very useful not only for oculoplastic surgeons but also for Brazilian ophthalmologists.

### THE CURRENT MOMENT

Currently, the Brazilian Society of Oculoplastic Surgery (SBCPO) occupies a prominent place among its peers, bringing 369 members associated with annuities. There are 17 Services currently available in Brazil dedicated to Fellowships, with 12 of them being in the southeast region. Several young oculoplastic surgeons excel and dominate the national scene. The scientific level of the society congresses and meetings raises to international standards. The Brazilian Society was recognized as a partner by the American Society in an agreement signed in June 2013 and by the European Society in October 2017. It is interesting to note that the Society has been held together and united in these 46 years. An analysis of the conference programs, courses, symposia, and congresses organized by boards has shown that the Eyelids, Lacrimal System, Orbit, and now Esthetics have been maintained as sisters and brothers from the same family. This union promotes the progress and strength of the Society on the national scene and, above all, promotes the power to defend its interests, particularly regarding fairer fees^(2)^.

The moment is observed with excitement and satisfaction to what has been conquered by the generations that succeeded in the command of the Society.

### RECOGNITION TO PRESIDENTS AND THEIR DIRECTORS

1975-1977 - Eduardo Jorge Carneiro Soares (MG)

1977-1979 - Evaldo Machado dos Santos (RJ)

1979-1981 - Eurípedes da Mota Moura (SP)

1981-1983 - Eduardo Jorge Carneiro Soares (MG)

1983-1985 - Sebastião Eloy Pereira (MS)

1985-1987 - Waldir Martins Portellinha (SP)

1987-1989 - Vicente Muniz de Carvalho (GO)

1989-1991 - Valênio Perez França (MG)

1991-1993 - Marilisa Nano Costa (SP)

1993-1995 - Waldir Martins Portellinha (SP)

1995-1997 - Roberto Caldato (SP)

1997-1999 - Hélcio Fortuna Bessa (RJ)

1999-2001 - Ana Rosa Pimentel (MG)

2001-2003 - Antônio Augusto Velasco e Cruz (SP)

2003-2005 - Ana Estela B. P. Santana( SP)

2005-2007 - Raquel Dantas (MG)

2007-2009 - Silvana Artioli Schellini. (SP)

2009-2011 - Suzana Matayoshi (SP)

2011-2013 - Ricardo Morchbacker (RS)

2013-2015 - Guilherme Herzog (RJ)

2015-2017 - Murilo Alves Rodrigues (MG)

2017-2019 - Roberto Murillo Limongi (GO)

2019-2021 - Patrícia Akaishi (SP)

### THE FUTURE

Hilton Rocha used to say “…a way to build is this one of recalling.”

A man at birth begins a relentless countdown. Every day, every month, and every year that passes are debts in the general accounts of his existence. The opposite happens with our Society. Every year is another in its history, incorporated to its heritage and contributing to consolidating it as a bank of knowledge and experience to be used for encouraging the growth of future generations. Such is life and the rotation is its law. Hilton Rocha would say, “We are all knelt down before a work that does not wither; on the contrary it germinates, grows, blooms.”
